# Factors influencing polymerase chain reaction outcomes in patients with clinically suspected ocular tuberculosis

**DOI:** 10.1186/1869-5760-4-10

**Published:** 2014-03-25

**Authors:** Praveen Kumar Balne, Rohit Ramesh Modi, Nuzhat Choudhury, Neha Mohan, Manas Ranjan Barik, Tapas Ranjan Padhi, Savitri Sharma, Satya Ranjan Panigrahi, Soumyava Basu

**Affiliations:** 1 L V Prasad Eye Institute, Hyderabad, India; 2 L V Prasad Eye Institute, Patia, Bhubaneswar, Odisha 751 024, India; 3 Department of Ophthalmology, Bangabandhu Sheikh Mujib Medical University, Dhaka, Bangladesh; 4 District Tuberculosis Center, Capital Hospital, Bhubaneswar, India

**Keywords:** Ocular tuberculosis, Polymerase chain reaction, Uveitis

## Abstract

**Background:**

Polymerase chain reaction (PCR) assay can be a useful method for definitive diagnosis in paucibacillary infections such as ocular tuberculosis (TB). In this study, we have evaluated factors affecting PCR outcomes in patients with clinically suspected ocular TB. Patients with clinically suspected ocular TB were investigated by PCR of aqueous or vitreous samples. Three control groups were also tested: group 1 included culture-proven non-tuberculous endophthalmitis, group 2 culture-negative non-tuberculous endophthalmitis, and group 3 patients undergoing surgery for uncomplicated cataract. PCR targeted one or more of following targets: *IS6110*, *MPB64*, and *protein b* genes of *Mycobacterium tuberculosis* complex. Multiple regression analysis (5% level of significance) was done to evaluate the associations between positive PCR outcome and laterality of disease, tuberculin skin test (TST)/interferon-gamma release assay (IGRA), chest radiography, and type of sample (aqueous or vitreous). The main outcome measures were positive PCR by one or more gene targets, and factors influencing positive PCR outcomes.

**Results:**

All 114 samples were tested for *MPB64*, 110 for *protein b*, and 88 for *IS6110. MPB64* was positive in 70.2% (*n* = 80) of tested samples, *protein b* in 40.0% (*n* = 44), and *IS6110* in only 9.1% (*n* = 8). DNA sequencing of amplicons from four randomly chosen PCR reactions showed homology for *M. tuberculosis* complex. Of the 80 PCR-positive patients, 71 completed a full course of antitubercular therapy, of which 65 patients (91.5%) had complete resolution of inflammation at final follow-up. Among controls, 12.5% (3 out of 24) in group 1 and 18.7% (6 out of 32) in group 2 also tested positive by PCR. No PCR-positive outcome was observed in control group 3 (*n* = 25). Multiple regression analysis revealed significant association of positive PCR outcome with bilateral presentation, but not with a positive TST/IGRA, chest radiography, or type of sample (aqueous/vitreous) used.

**Conclusions:**

Careful selection of gene targets can yield high PCR positivity in clinically suspected ocular TB. Bilateral disease presentation but not any evidence of latent systemic TB influences PCR outcomes. False-positive results may be seen in ocular inflammation unrelated to ocular TB.

## Background

Ocular tuberculosis (TB) is the most common cause of infectious uveitis in high endemic countries [1]. However, the paucibacillary nature of this disease limits its detection by conventional techniques like smear microscopy and culture. Nucleic acid amplification tests (NAATs) like polymerase chain reaction (PCR) can therefore be a useful alternative for definitive diagnosis of this condition [2].

PCR-based diagnosis of any form of extrapulmonary TB is challenging due to the paucibacillary nature of specimens, lack of adequate sample volumes, non-uniform distribution of bacteria in such specimens, presence of PCR inhibitors, and lack of proper gold standard for evaluation of its diagnostic potential [3]. Past attempts at PCR for ocular TB have yielded low positivity rates ranging from 33.3% for retinal vasculitis to 66.6% for panuveitis [4]. Not surprisingly, PCR has not gained popularity in the diagnosis of ocular TB despite being first described nearly two decades ago [5]. As a result, much of clinical practice as well as existing literature is based on presumed diagnosis of ocular TB. Such presumed diagnosis may potentially lead to both under- and overdiagnosis of the condition, exposing the patient to unprotected steroid therapy or toxic antitubercular therapy (ATT), respectively.

The initial PCR studies for ocular TB were based on single-target PCR, typically with *IS6110* as the gene target [4,5]. About 2 years ago, multi-target PCR was shown to have high specificity (100%) and sensitivity (73.68%) in a series of 19 patients with suspected ocular TB (Sharma K et al., Novel multiplex polymerase chain reaction for diagnosis of intraocular tuberculosis. Poster at: AAO Annual Meeting, Orlando, 2011). The authors had used three gene targets (*IS6110*, *MPB64*, and *protein b*) since the commonly used target, *IS6110*, was reported to be absent in up to 11% of patients in certain populations [6]. The encouraging results of this report prompted us to initiate routine use of PCR in the diagnosis of ocular TB. The present study is a retrospective analysis of the PCR outcomes, clinical characteristics, and results of treatment in patients with clinically suspected ocular TB, who were investigated with PCR at our institute. We have attempted to determine the factors that could have influenced PCR outcomes in our patients.

## Methods

The study was approved by the institutional review board of L V Prasad Eye Institute and adhered to the tenets of the Declaration of Helsinki. We included all patients, clinically suspected to have ocular TB and investigated with PCR, between November 2011 and February 2013 at our institute. The clinical diagnosis was based on the presence of any one or more of the following - granulomatous anterior uveitis, intermediate uveitis, retinal vasculitis, serpiginous-like choroiditis, focal or multifocal choroiditis, and panuveitis - and exclusion of other uveitic entities, which could cause similar clinical presentation. Grades of inflammation and criteria for response to treatment were based on Standardization of Uveitis Nomenclature (SUN) working group recommendations [7]. All patients received tuberculin skin test (TST) and/or interferon-gamma release assay (IGRA) and at least one form of chest radiography (plain X-ray/high-resolution computed tomography). Positive TST was defined as induration ≥10 mm after subcutaneous injection of 5 tuberculin units in immune-competent patients. Physician referral was done to rule out any other focus (pulmonary/extrapulmonary) of TB.

### Sample collection

We included patients who had anterior chamber cells, along with clinical signs suggestive of ocular TB. Around 150 to 200 μl of aqueous humor was collected by anterior chamber paracentesis under strict aseptic precautions. In selected patients (*n* = 20), like those receiving therapeutic vitrectomy for non-resolving intermediate uveitis, vitreous samples were used for PCR.

### DNA extraction

DNA was extracted from all the samples using QIAamp DNA mini kit (Qiagen, Hilden, Germany) tissue protocol, with the following modifications: an enzymatic digestion step with 30 mg/ml lysozyme added to the tissue lysis buffer and incubated at 37°C for 1 h followed by adding 20 μl of proteinase K and 200 μl of lysis buffer and incubated at 56°C for 1 h. DNA was purified as per manufacturer's recommendations through a minispin column, and 50 μl of elution buffer was used to elute the DNA. Extracted DNA was stored at -20°C until PCR testing.

### PCR

Multi-target PCR targeting *IS6110*, *MPB64*, and *protein b* in *Mycobacterium tuberculosis* complex was standardized as described previously [8]. Details of the primer sequences were as mentioned in Table 1. Amplification was performed in a 50-μl reaction mixture including 10 pmol of each primer, 200 μM each deoxynucleoside triphosphate, 1.5 mM MgCl_2_, 10× PCR buffer without MgCl_2_, 1 unit of Taq DNA polymerase, and 10 μl of template. The PCR cycles consisted of an initial denaturation step at 94°C for 5 min, 40 cycles of denaturation at 94°C for 1 min, annealing at 65°C for 20 s with an extension at 72°C for 30 s, and a final extension at 72°C for another 10 min. Double autoclaved Milli-Q water (Millipore Co., Billerica, MA, USA) was used as negative control, and *M. tuberculosis* H37Rv DNA was used as a positive control. Both controls were included in each PCR run. After PCR, the amplified products were electrophoretically resolved in an ethidium bromide-stained 1.5% agarose gels in 1× Tris acetate EDTA buffer and visualized under UV transillumination. Care was taken to avoid contamination by doing all procedures like isolation of DNA, PCR master mixture preparation, addition of template, PCR, and gel electrophoresis in separate rooms.

**Table 1:** Polymerase chain reaction primer sequences and their amplicon sizes

**Primer name**	**Sequence (5′-3′)**	**Amplicon size**
IS 6110 F	CCTGCGAGCGTAGGCGT	123 bp
IS 6110 R	CTCGTCCAGCGCCGCTTCGG	
MPB64 F	TCCGCTGCCAGTCGTCTTCC	240 bp
MPB64 R	GTCCTC GCG AGT CTA GGC CA	
Protein b F	ACCACCGAGCGG TTCGCCTGA	419 bp
Protein b R	GATCTGCGGGTCGTCCCAGGT	

### Analytical sensitivity and specificity

The analytical sensitivity of PCR was determined using different concentrations of *M. tuberculosis* H37Rv DNA and was found to be 250 fg per reaction (50 copies/reaction). The analytical specificity of PCR was verified using DNA from different microorganisms (bacterial, fungal, viral, and parasitic) and human leukocytic DNA. No amplification was observed with DNA other than *M. tuberculosis* H37Rv.

### Detection limit for individual primers in multi-target PCR assay

Aqueous humor from patients undergoing cataract surgery was spiked with 10^6^ colony forming units (CFU) of *M. tuberculosis* per 100 μl of sample. It was then diluted to achieve 10^5^, 10^4^, 10^3^, 10^2^, 10, and 5 CFU per 100 μl of sample, respectively. DNA was extracted from each of the samples, and multi-target PCR was performed for them. This also helped in ruling out PCR inhibition in ocular samples.

### DNA sequencing

Amplicons from four PCR reactions were randomly chosen for sequencing to demonstrate homology with *M. tuberculosis* complex. Sequencing was performed with fluorescence-labeled dideoxynucleotide terminators using an ABI 3130 Xl automated sequencer, following the manufacturer's instructions (PE Applied Biosystems, Foster City, CA, USA). The sequences were analyzed and identified using the MEGABLAST search program of the GenBank database.

### Controls

The study had three control groups. Group 1 had 24 vitreous samples collected from patients with culture-proven non-tuberculous endophthalmitis (disease controls), group 2 had 32 vitreous samples from patients with culture-negative endophthalmitis (clinically non-tuberculous, also disease controls), and group 3 had 25 aqueous samples collected from patents who underwent cataract surgery (negative controls).

### Statistical analysis

Descriptive statistics was used to calculate PCR positivity rate in cases and controls. Multiple regression analysis was used to assess association between positive PCR outcome and laterality of disease, TST/IGRA, chest radiograph, and duration of current attack of uveitis (InStat statistical software version Win 3.0x, GraphPad Software, Inc. CA, USA).

## Results

A total of 114 ocular fluid samples from 114 subjects with clinically suspected ocular TB were tested, and positive PCR outcomes were obtained in 70.2% (*n* = 80) patients. The baseline characteristics of these patients are given in Table 2. Majority of the tested samples (82.5%; *n* = 94) were aqueous taps, while the remainder consisted of vitreous samples. The most common clinical presentation among our patients was retinal vasculitis (33.3%; *n* = 38) followed by panuveitis (21.9%; *n* = 25). Patients with retinal vasculitis, panuveitis, or anterior uveitis had a higher PCR positivity rate, as compared to intermediate uveitis and serpiginous-like choroiditis (data not shown). Significantly, nearly half the total patients (45.6%; *n* = 52) did not have any immunological evidence of previous tuberculous infection (tuberculin test/IGRA), while nearly four fifths (79.8%; *n* = 91) did not have any radiological evidence of healed TB. Similar proportions were noted in the PCR-positive group: 43.8% (*n* = 35) had negative tuberculin test/IGRA, and 78.7% (*n* = 67) had normal chest radiograms. None of the patients had any evidence of active systemic TB, possibly because only such patients were selected for the PCR test.

**Table 2:** Baseline characteristics of patients undergoing polymerase chain reaction for diagnosis of ocular TB

**Baseline characteristics**		**Number of patients (%)**
Age	33.7 ± 12.7 years	
Gender	Females	43 (37.8)
	Males	71 (62.3)
PCR outcome	Positive	80 (70.2)
	Negative	34 (29.8)
Diagnosis	Anterior uveitis	18 (15.8)
	Intermediate uveitis	15 (13.2)
	Retinal vasculitis	38 (33.3)
	Multifocal serpiginoid choroiditis	14 (12.3)
	Panuveitis	25 (21.9)
	Others	4 (3.5)
Laterality	Bilateral	56 (49.1)
	Unilateral	58 (50.9)
Tuberculin skin test/Quantiferon TB Gold Test	Negative	52 (45.6)
	Positive	62 (54.4)
Chest radiography	Normal	91 (79.8)
	Abnormal	23 (20.2)
Sample	Aqueous	94 (82.5)
	Vitreous	20 (17.5)

Of the total 114 samples, the initial 88 samples were tested by all three targets (*IS6110*, *MPB64*, and *protein b*) and 67 (76.1%) were found to be positive. Of these, *MPB64* was positive in all 67 samples, *protein b* in 41 samples, and *IS6110* in 8 (9.1%) samples. All *IS6110* positives were also positive by *MPB64* and *protein b*. Based on these results, we discontinued using the *IS6110* gene target, and the next 22 samples were tested by PCR targeting *MPB64* and *protein b* only and 9 were found to be positive. Again, *MPB64* was positive in all nine samples and *protein b* in three of these nine samples. The last four samples included in this study were tested only with *MPB64*, and all were positive.

**Figure 1:**
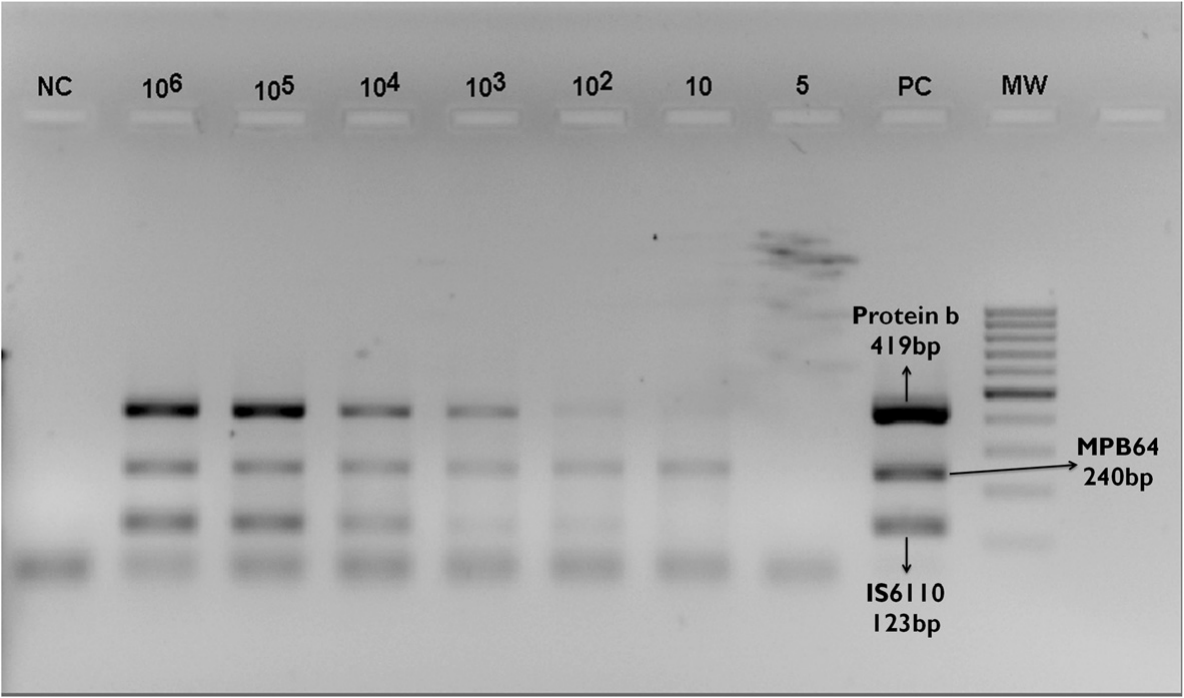
**Detection limits of individual primers in multi-target PCR assay.** Agarose gel (2%) electrophoresis of multi-target PCR assay for serial dilutions of H37Rv *Mycobacterium tuberculosis*, showing detection limit for *protein b* and *IS6110* at 102 colony forming units (CFU) and for *MPB64* at 10 CFU. No amplification was seen at 5 CFU for any of the primers.

The detection limit of *MPB64* primers were found to be 10 CFU/100 μl, while that for *protein b* and *IS6110* were 10^2^ CFU/100 μl (the band for *IS6110* appearing fainter than *protein b* at this dilution, Figure 1). No amplification was observed at 5 CFU dilution. DNA sequencing of the four PCR amplicons that were tested showed homology with the *M. tuberculosis* complex.

Of the 80 patients with positive TB PCR outcomes, 77 agreed for treatment with anti-TB therapy (ATT) and adjunctive corticosteroids, and 71 completed a 6-month course of ATT. Of these 71 patients, 65 (91.5%) had resolved inflammation at final follow-up, while the rest (*n* = 6; 8.5%) had persistent or recurrent episodes of inflammation.

The PCR outcomes in the control groups are detailed in Table 3. In group 1 (culture-positive endophthalmitis), 3 of 24 samples (12.5%) were positive for *M. tuberculosis* complex DNA by multi-target PCR. In group 2 (culture-negative endophthalmitis, *n* = 32), six (18.75%) had positive PCR outcomes. One PCR-positive sample in this group was also smear positive for fungus*.* No PCR amplification was observed in group 3 (cataract, *n* = 25).

**Table 3:** **Baseline characteristics of control groups 1 and 2 (MTB, *Mycobacterium tuberculosis*; PCR, polymerase chain reaction)**

	**Group 1 (culture-positive, *n*** **= 24)**	**Group 2 (culture-negative, *n*** **= 32)**
Postoperative endophthalmitis	7	16
MTB PCR positive	2	4
MTB PCR negative	4	12
Posttraumatic endophthalmitis	10	13
MTB PCR positive	0	3
MTB PCR negative	10	10
Endogenous endophthalmitis	7	3
MTB PCR positive	1	0
MTB PCR negative	6	3

On multivariate regression analysis, only bilateral presentation of uveitis appeared to favor a positive PCR outcome in our study (Table 4). Positive TST/IGRA or positive chest radiography did not appear to influence the PCR outcome. Also, there was no difference in PCR outcomes between using aqueous or vitreous samples.

**Table 4:** Multivariate regression analysis of factors influencing outcomes of polymerase chain reaction

	**Univariate *p *** **value**	**Wald**	**Multivariate *p *** **value**	**Odds ratio**	**95% confidence interval**
Bilaterality	0.0077	7.540	0.006	3.419	1.422 to 8.220
Tuberculin skin test/Quantiferon TB Gold Test	0.5461	0.164	0.685	0.661	0.352 to 1.988
Chest radiography	0.8007	0.530	0.466	0.836	0.217 to 2.014
Aqueous (vs. vitreous) sample	0.7890	0.313	0.576	0.721	0.229 to 2.270

## Discussion

PCR offers the only opportunity for definitive diagnosis in majority of cases of ocular TB. Yet, its application in clinical practice has been limited mostly due to poor positivity rates. In this study, we have reported high PCR positivity (70.6%) in ocular TB, with recently introduced modifications in the PCR technique. Our PCR outcomes were validated by DNA sequencing of the PCR amplicons and beneficial effect of ATT in majority (91.5%) of the PCR-positive patients. Further, we have analyzed the factors influencing the PCR outcomes in our ocular TB patients.

Evidently, the key factor behind the high PCR positivity (70.6%) in our patients was the selection of gene targets. Initially, we used multi-target PCR with three different gene targets but later narrowed down to a single target - *MPB64* - since it was present in all PCR-positive samples. In contrast, the traditionally used gene target *IS6110* was found in only 9.1% of the samples. This unexpected result could be attributed to two factors: differences in primer efficiency in multi-target PCR conditions and regional variations in mycobacterial strains and, thereby, gene targets. As shown in Figure 1, the bands for *protein b* and *IS6110* were barely visible at a dilution of 10^2^ CFU/100 μl of the sample, while that for *MPB64* was significantly brighter even at 10 CFU/100 μl dilution. Since the template for this PCR assay (Figure 1) was obtained from a known concentration and strain of *M. tuberculosis*, such difference in detection limits for individual primers would have resulted from the difference in primer efficiency in multi-target PCR conditions. Thus, *IS6110* and/or *protein b* may not have been detected despite being present in our samples. Although these two primers differ in detection limit from *MPB64*, only by a factor of 10 (one log unit), such a difference can be significant in extreme paucibacillary conditions such as ocular TB. In a recent report from north India that used the same primers and PCR conditions, two of seven samples were positive only for *MPB64* though they also had one sample positive only for *IS6110*[9]. Interestingly, such gross difference in *MPB64* and *IS6110* positivity was not seen when the same multi-target PCR assay was performed in TB meningitis patients [8]. We speculate that higher bacillary load in TB meningitis as compared to ocular TB could have accounted for this variation.

There is also a possibility of *M. tuberculosis* strain variations in our part of the country, or in infections affecting the eye, such that *IS6110* may have been absent or present in reduced copy numbers in our samples. An earlier study from south India had highlighted the significantly higher sensitivity for *MPB64* than *IS6110* in PCR on epiretinal membranes from Eales' disease patients [10]. Based on these results, we propose that *MPB64*-based uniplex PCR is sufficient for diagnosis of majority of ocular TB cases in India. Similar ‘region-specific’ PCR can be planned for other geographic regions based on positivity of specific gene targets. For example, in the USA, *IS6110* is absent in only 1% of *M. tuberculosis* strains and can thus be an ideal gene target for PCR [11].

We also tried to investigate the role of other possible factors influencing PCR outcomes by multiple regression analysis. We found that only bilateral disease presentation favored a positive PCR outcome, although infectious ocular diseases are typically associated with unilateral presentation. Interestingly, a recent report noted bilateral presentation in about 63% of 105 patients with tubercular multifocal serpiginoid choroiditis [12]. More importantly, positive immunological tests (TST/QFT) or chest radiography did not influence PCR outcomes in our study. This correlates with an earlier report of histopathologically proven ocular TB, in which 40% of patients had negative TST and 57% had normal chest films [13]. Also, reactivation of tuberculosis at extrapulmonary sites is known to occur without active pulmonary TB, in 15% cases [14].

Besides the above factors, we believe that the diagnostic yield of PCR in our study was improved by our case selection, based on previously described ocular signs [15]. A recent systematic review on the role of NAATs in smear-negative pulmonary TB showed that a positive NAAT was found most useful in ruling in pulmonary TB when the pretest probability was at least intermediate (between 30% and 40%) [16]. However, a negative NAAT in this group was not useful in ruling out the disease. If extrapolated to ocular TB, a positive PCR if considered with clinical signs and other investigations can help in reaching the final diagnosis, though a negative PCR would not rule out the disease.

We could not determine the diagnostic sensitivity and specificity of PCR in our study due to lack of an appropriate gold standard (culture/microscopy). Previous studies have considered clinical diagnosis as the ‘gold standard’ but that remains highly debatable [9,10]. There is another important caveat that needs to be addressed for wider acceptance of diagnostic PCR, especially in TB-endemic countries. As seen in our control groups 1 and 2, a significant number of patients with ocular inflammation unrelated to TB may have positive PCR for TB. All except one of the PCR-positive patients in control groups had either postoperative or posttraumatic endophthalmitis. The only PCR-positive endogenous endophthalmitis patient was culture positive for *Aspergillus flavus*. Hence, we are convinced that all the positive PCR outcomes in the control groups represented false-positive outcomes. In the earlier report from north India, none of the six controls with intraocular inflammation unrelated to TB had positive PCR for TB [9]. Though we had a much larger control group with intraocular inflammation (*n* = 56), the high incidence of false-positive outcomes (16.1%) in this group needs further explanation. It is known that *M. tuberculosis* can persist in several cell types and sites in the body, without any lung involvement, especially in highly endemic populations [17,18]. Thus, it is possible that inflammatory cells present in aqueous or vitreous sample may have harbored the bacilli or its DNA, giving rise to false-positive results. Future studies with reverse transcriptase PCR for mycobacterial mRNA may help in identifying viable bacilli in the eye, thereby providing a more definitive diagnosis [19].

## Conclusions

To conclude, careful selection of gene targets can help us achieve high PCR positivity in clinically suspected ocular TB patients. Immunological or radiological evidence of latent TB does not influence the PCR outcome. However, false-positive PCR in patients with non-TB ocular inflammation remains a challenge that needs to be resolved.

## Competing interests

The authors declare that they have no competing interests.

## Authors’ contributions

SB conceived, designed, analyzed and interpreted the data, and drafted and revised the manuscript. PKB performed the data acquisition, analysis, and interpretation, and drafted and revised the manuscript. RRM performed the data acquisition, analysis, and interpretation, and revised the manuscript. NM performed the data analysis and interpretation, and revised the manuscript. NC, MRB, TRP, and SRP performed the data acquisition and revised the manuscript. SS performed the data analysis and interpretation and revised the manuscript. All authors read and approved the final manuscript.

## Authors’ information

NC participated in the project during her fellowship at L V Prasad Eye Institute, Bhubaneswar. PKB and SS were also at L V Prasad Eye Institute, Bhubaneswar, during the period of study.
